# The Time Is Ripe: Thinking about the Future Reduces Unhealthy Eating in Those with a Higher BMI

**DOI:** 10.3390/foods9101391

**Published:** 2020-10-01

**Authors:** Betty P. I. Chang, Maria Almudena Claassen, Olivier Klein

**Affiliations:** 1 European Food Information Council, 1000 Brussels, Belgium; 2 School of Psychology, University of Glasgow, G12 8QQ Glasgow, Scotland; MariaAlmudena.Claassen@glasgow.ac.uk; 3 Centre for Social and Cultural Psychology, Free University of Brussels, 1050 Brussels, Belgium; Olivier.Klein@ulb.be

**Keywords:** time perspective, temporal perspective, self-regulation, time preference, eating, BMI, obesity

## Abstract

Research suggests that being oriented more towards the future (than the present) is correlated with healthier eating. However, this research tends to be correlational, and thus it is unclear whether inducing people to think about their future could increase healthy eating. Therefore, we investigated whether inducing people to think about their lives in the future versus the present would influence their intake of healthy (muesli) and unhealthy (Maltesers) food. Across two experiments, the effect of thinking about the future versus the present interacted with participants’ body mass index (BMI) to influence their consumption of unhealthy food, but no reliable effects were found for the consumption of healthy food. Among individuals with a higher BMI, thinking about their lives in the future resulted in lower consumption of the unhealthy food compared to thinking about their lives in the present. However, this effect was reversed for those with a lower BMI. In Experiment 2, we found no evidence that this effect was due to reduced impulsivity (as measured by a delay discounting task and a stop-signal task). This suggests that thinking about the future can reduce unhealthy eating among heavier people.

## 1. Introduction

Although healthy eating is important for physical [1] and mental health [2,3], people vary widely in the extent to which they are motivated to eat healthily. One individual difference that has been associated with the healthiness of dietary habits is the extent to which people focus on the present or the future [4,5,6,7]. This factor is known as ‘time perspective’ (a.k.a. temporal perspective, temporal/time orientation, e.g., [8,9]). An important aspect of time perspective is the extent to which people take into account the future consequences of their actions when engaging in a certain behavior (Consideration of Future Consequences, [6]). Those who are relatively more oriented towards the future are considered to have a future time perspective and tend to value long-term benefits over more immediate gratification [6,10]. Those who are more oriented towards the present are considered to have a more present-time perspective and are more likely to be motivated by short-term over long-term consequences [6,10]. The consideration of future consequences (CFC) is measured using the Consideration of Future Consequences Scale [6], with separate subscales for the CFC-Immediate, and the CFC-Future. Most studies consider CFC as a unidimensional construct, with scores on the CFC-Immediate subtracted from scores on the CFC-Future.

Research shows that more present-oriented individuals prefer an immediate, smaller reward (e.g., receiving 5 dollars today) over a larger reward in the future (e.g., receiving 10 dollars next week), whereas the opposite is true of those who are more future-oriented, e.g., [11,12]. This phenomenon is known as delay discounting, with present-oriented individuals described as showing greater delay discounting (see [13], for a review). Greater delay discounting is thought to represent greater impulsivity, as waiting would result in a larger payoff.

Studies have shown a relationship between, on the one hand, various measures of time perspective, and, on the other hand, (un)healthy eating and its correlates. For example, one study measured CFC as a unidimensional construct in a representative sample of 2000 Dutch households [5]. Participants who had a stronger present orientation had a small but significant increased risk of obesity, controlling for age, gender, and education. A similar result has been found in a US urban population [14]. When CFC was considered as a two-factor construct in a sample of 800 English participants, a stronger present-time perspective as measured on the CFC-Immediate subscale was related to a higher body mass index (BMI) [4], and a similar effect has been found with less healthy eating in the Netherlands [7]. Less consistent results have been found with Zimbardo’s Time Perspective Inventory [8], with very few studies finding a relationship between time perspective and BMI [15], whereas others tend not to show any relationship [14,16].

The relationship between being more present-oriented and eating less healthily (or being more future-oriented and eating more healthily) is thought to occur because healthy eating often involves eschewing immediate rewards for long-term benefits, such as giving up the instant pleasure of eating indulgent and/or readily-available food in favor of food that is more healthy. Those who are more present-oriented, however, are more likely to value the immediate gratification that unhealthy, fast food offers. Consistent with this, studies show that those who exhibit greater delay discounting are also more likely to have unhealthy eating patterns, a higher percentage body fat, or a greater BMI, particularly in women (for reviews, see [17,18,19]).

However, it is unclear whether people’s time preferences directly shape their eating habits, or whether there are other reasons that (un)healthy eating is associated with time perspective. It may be that conditions that facilitate (un)healthy eating, such as growing up in a household with a relatively (low) high socioeconomic status [20], also foster a (shorter) longer-term perspective [21]. In this way, time perspective may be associated with eating habits through a third variable. Indeed, research suggests that, at least in the US, the healthiest diet is slightly more expensive than the unhealthiest diet [22].

In order to test whether thinking about the future leads to healthier eating, Dassen and colleagues [23] experimentally manipulated whether hungry participants thought about the future or the past. Those in the future condition were instructed to write either about events that had been planned or could happen in the future, from two weeks to six months’ time. Those in the past condition wrote about events they had experienced from one week to one month ago. Participants in both conditions were further subdivided so that they wrote either about unspecified events or they wrote about food-related events. Participants were then given a range of sweet, unhealthy snacks to freely consume. They then completed a delay discounting task in which they were asked to make a series of choices between an immediate monetary reward or a larger, delayed monetary reward. Although participants in both the food-related and unspecified future condition showed a greater preference for the delayed reward (i.e., showed less delayed discounting), only those who thought about food-related events in the future consumed fewer calories than those who thought about food-related events in the past. There was no difference between participants who thought about events that would occur from two weeks to six months’ time.

However, because there was no control condition in the study of Dassen and colleagues [23], it is not clear whether thinking about food in the future decreases consumption or thinking about food in the past increases consumption. Furthermore, although it has been demonstrated that thinking about the past and the future can produce similar effects on decision-making [24], thinking about a distant future/past produces decisions that are less influenced by current mood than thinking about a near future/past. Thus, because Dassen and colleagues [23] asked participants in their study to think about an event that occurred in the range of 2 weeks to 6 months before/after the present time, differences between their future and past conditions could be due to differences in the time horizon between the conditions. That is, participants in the past condition may have recalled events in the near past, whereas those in the future condition may have thought of events in the distant future.

The association between a present time preference and unhealthy eating [7], or BMI [4], is clearer than the evidence of a relationship between future time preference and BMI [5]. Hence, examining whether inducing a present-time orientation increases consumption is critical to the investigation of whether manipulating time perspective influences healthy eating. Therefore, building upon the research of Dassen and colleagues [23], we aimed to examine if manipulating whether individuals think about the future versus the present affects food consumption. To examine which condition could be responsible for any possible differences between the two time perspectives, we also included a control condition in which participants summarized the last film they saw. Unlike Dassen and colleagues, who gave participants a future time horizon of 2 weeks to 6 months, we asked participants in the future condition to project 10 years from the present, as previous research examining future time perspective has adopted such a time frame [25,26,27].

Because we included a present-time perspective condition, it was not possible to follow the manipulation of Dassen and colleagues [23] and ask participants to think about a food-related event. Instead, we asked participants to write either about themselves and their life in the present, or how they imagined themselves and their life 10 years in the future. Nor did we ask participants to fast 2 h before participating in the experiment, as Dassen and colleagues [23]. Instead, we measured participants’ level of hunger and considered it as a potential moderator in our study, as previous studies have shown hunger to moderate the effects of manipulations that influence eating e.g., [28,29,30]. Finally, unlike Dassen and colleagues [23], who only offered participants unhealthy food, we offered participants both healthy and unhealthy food options, as we wished to examine whether the effects of manipulating time perspective were specific to unhealthy food, or whether they generalized to healthier food.

We hypothesized that participants in the future condition would consume less unhealthy food than participants in the present condition. However, we were agnostic as to whether there would be differences between the two conditions in the consumption of healthy food. If the effects of reduced eating are specific to unhealthy food, this suggests that the mechanism underlying the effect of time perspective on eating is based on the consideration of consequences, because it is sensitive to the positive/negative outcomes of consumption. However, if the effects of reduced eating apply to both healthy and unhealthy food, this suggests that the effects of time perspective may affect appetite more generally, rather than being based on the consideration of consequences.

### Pilot Study

Because there are several measures that are related to the construct of time perspective, we first conducted a pilot study with several measures of time perspective-related constructs and eating-related measures. The aim of this study was to examine which constructs were correlated with self-reported BMI and healthy and unhealthy eating. The results of this study would then be used to design subsequent experiments investigating the effect of manipulated time perspective on healthy and unhealthy consumption. Ethical approval for the studies in this paper were obtained from the ethics committee of the Faculty of Psychological and Educational Science of the Free University of Brussels (ULB), under the approval code 060/2015.

## 2. Materials and Methods

Ninety-one participants (35 female, 2 did not provide their sex; *M*_age_ = 28.80, *SD* = 6.04) completed a questionnaire online via the crowdsourcing website Prolific (https://prolific.ac/), using the survey platform Qualtrics (https://www.qualtrics.com/). They were paid £1.25 for their participation. The questionnaire consisted of the following time perspective-related measures:

a) Zimbardo’s Time Perspective Inventory (ZTPI; [8]). Specifically, they completed the subscales of Present-Hedonistic (α = 0.77, e.g., “I take risks to put excitement in my life.”), Present Fatalistic (α = 0.80, e.g., “My life path is controlled by forces I cannot influence.”), and Future (α = 0.73, e.g., “I make lists of things to do.”).
b) Consideration of Future Consequences Scales [6]: Immediate subscale (α = 0.79, e.g., “I only act to satisfy immediate concerns, figuring the future will take care of itself.”), and Future subscale (α = 0.84, e.g., “I consider how things might be in the future, and try to influence those things with my day to day behavior.”).
c) Future Self-Continuity Scale [31]. This scale measures how similar people perceive their current self will be to their self 10 years in the future. Participants are presented with circle pairs with varying degrees of overlap, with one circle in each pair representing their current self, and the other circle representing their future self. Participants are asked to choose which circle pair best describes how similar and how connected they feel to their future self 10 years from now.

In addition, participants reported the frequency with which they ate healthy (e.g., fruits, vegetables, pasta, and rice, α = 0.73) and unhealthy food (e.g., fried food, desserts, and processed snack foods, α = 0.59), on a scale from 1 = *0 times per week*, to 5 = *6 or more times per week*. We measured the extent to which participants regulated their food intake in relation to their weight using the restrained eating scale of the Dutch Eating Behaviour Questionnaire (DEBQ; [32]; α = 0.91). They also reported their height and weight, with which we calculated their body mass index (BMI).

### 2.1. Experiment 1

#### 2.1.1. Participants

Sixty-five participants (*M_age_* = 21.12, SD = 3.02; 71% female) were recruited from a Belgian university. They were all students of the university, ranging from undergraduate to postgraduate level, and were paid 5 euros for their participation. The participants were randomly assigned to one of three experimental conditions.

#### 2.1.2. Design

The design consisted of a between-participant factor of condition with 3 levels (present-self/future-self/control) × a within-participant variable of outcome type with 2 levels (healthy vs. unhealthy food). Hunger, restrained eating, and BMI were measured as continuous moderators.

#### 2.1.3. Measures

The following measures were taken:

a) Consideration of Future Consequences Scales [6]. We used the validated French translation of this scale [33], which included the CFC-Immediate subscale (e.g., “I only act to satisfy immediate concerns, figuring the future will take care of itself”; α = 0.85), and the CFC-Future subscale (e.g., “I think it is more important to perform a behavior with important distant consequences than a behavior with less important immediate consequences”). Because the reliability of the full CFC-Future subscale was low (α = 0.50), we removed the least reliable question (“I am willing to sacrifice the immediate happiness or well-being in order to achieve future outcomes”), in order to increase the reliability of the subscale (α = 0.86). The scale ranged from 1 = *does not correspond at all to me to* 7 = *corresponds completely with me*. An overall CFC score was calculated by subtracting the CFC-Immediate subscale from the CFC-Future subscale. Thus, higher scores indicated a stronger future orientation.
b) Mood was assessed with the Positive and Negative Affect Schedule (PANAS; [34]), consisting of two 10-item scales assessing the extent to which participants feel positive (α = 0.84) and negative affect (α = 0.81). Responses ranged from 1 = *Not at all* to 5 = *Extremely*.
c) Participants’ levels of restrained eating was assessed with the restrained eating subscale of the DEBQ [32]. This subscale consists of five items such as “Do you watch exactly what you eat?” (α = 0.76). Responses ranged from 1 = *Never* to 5 = *Very often*.
d) Hunger levels were assessed with two items that were embedded in the PANAS, which asked participants to what extent they felt satiated and to what extent they felt hungry (α = 0.67). Responses ranged from 1 = *Not at all* to 5 = *Extremely*.
e) Participants’ BMI was calculated using measures of height (m) and weight (kg) taken by the researchers.

#### 2.1.4. Procedure

Participants were told that the study involves examining the link between language style (via a writing task) and sensory perception (via a taste test). Half of the participants then completed the CFC Scale (the other half completed it at the end of the study). They were then asked to write about how they imagine their future self and situation to be in 10 years’ time (future-self condition), to describe their current self and situation (present-self condition), or to describe the last film they saw (control condition). Participants were told that they had up to 12 min to complete this task. Following this, participants completed the PANAS with two embedded items measuring their level of hunger. Then they were presented with one bowl of unhealthy food: Maltesers (chocolate-covered malted milk biscuit balls) and one bowl of healthy food: muesli. They were asked to take as much of each food as they liked (with serving spoons, on a paper plate), and to rate the food on the dimensions of sweetness, saltiness, bitterness, and textural elements. Participants were offered a glass of water for this task, and when they ate, they were faced away from the experimenter. Then participants were asked what they thought the purpose of the study was, with none being able to correctly guess the true purpose. Lastly, they completed the Restraint questionnaire, and their height and weight (without shoes) were measured.

### 2.2. Experiment 2

Experiment 2 was conducted to see if the results of Experiment 1 could be replicated. We also sought to test whether changes in impulsivity could be responsible for the underlying effects. More specifically, we examined whether the effect of time perspective on food consumption is mediated by impulsivity. We assessed two components of impulsivity: delay discounting and inhibitory ability, which may be affected by the time perspective manipulation of Experiment 1. We used a general, money-related discounting task, and a food-specific discounting task. The inhibition task we used was the stop-signal task [35].

We hypothesized that the effect of the Future (vs. Present) condition on Malteser consumption would be mediated by: (a) monetary discounting, (b) food discounting, and (c) inhibitory ability. That is, participants in the future condition will discount money and food less, and will have better inhibition, compared to participants in the present condition. Moreover, we predicted that higher impulsivity on these measures would predict Malteser consumption.

#### 2.2.1. Participants

One-hundred-and-thirty-six participants (*M_age_* = 20.50, *SD* = 4.55; 75% female) were recruited from a Belgian university. They were all first-year psychology students who performed the study in exchange for course credits.

#### 2.2.2. Design and Procedure

The design and procedure of Experiment 2 was the same as that of Experiment 1 except that, after participants performed the bogus taste test, they completed two additional tasks before filling in the dietary restraint subscale, the CFC, and questions about their socioeconomic status. These two tasks were:

a) a money and food discounting task (Money Choice Questionnaire [35], and Food Choice Questionnaire [36]); and
b) the stop-signal task [37] measuring inhibitory ability.

#### 2.2.3. Measures

*Discounting tasks.* Money delay discounting was measured using the Money Choice Questionnaire (MCQ; [35]). This task consisted of 27 hypothetical choices between receiving an immediate smaller amount of money versus waiting for a larger amount of money. The amounts as well as the time delays varied in sizes. For instance, participants chose between receiving $27 now or waiting 21 days and receiving $50.

Similarly, food delay discounting was assessed with the Food Choice Questionnaire (FCQ; [36]). This consisted of 27 hypothetical choices between immediate smaller amounts of bites of food or delayed larger amounts of bites of food, again with varying amounts of bites and time delays. Participants saw a one cm^3^ white cube and were asked to imagine this to be a bite of their favorite food. They were told that the choices were hypothetical but they were asked to imagine that they would actually receive the reward. They were asked to choose between, for instance, 15 bites of their favorite food now or 35 bites in eight hours.

For both types of rewards, the pattern of responses of the participants was transformed to three discounting scores *k* for each size of reward (small, medium, large). For money, these scores were calculated using the automated scorer created by Kaplan et al. [38] and for food with a scoring sheet provided by the authors of the scale [36]. Discounting scores *k* range from 0.00016 to 0.25 for money, and 0.0252 to 0.854 for food, with lower scores representing lower impulsivity. For the analyses, discounting scores were transformed to their natural log(ln) to normalize the distribution [39].

#### 2.2.4. Stop-Signal Task

The stop-signal task measures the inhibition of prepotent responses by presenting participants with intermixed trials of a go task and a stop task. For the go tasks, participants are required to respond as quickly as possible to a predetermined stimulus (a left or right pointing arrow, in this case), the go trial, but to withhold any response when a stop signal (an audio tone in this case) is presented. In this case, participants responded by pressing a key (‘B’ in response to the left arrow, and ‘N’ in response to the right arrow). Participants had a limited window of 500 ms in which to respond. If the go process (i.e., execution of the prepotent response) finishes before the stop process (i.e., the inhibition of the prepotent response), then inhibition is not successful.

Participants received 8 practice trials and 192 test trials, with the stop signal occurring on a minority of trials. Thus, the participant forms a prepotent response pattern of completing the go task on every trial, but on a minority of trials has to inhibit that prepotent response. In the practice trials only, participants received feedback when they did not successfully inhibit their response to the stop signal—i.e., they saw the message “You should not have pressed a key”. The stop-signal task was programmed in E-prime (https://www.pstnet.com).

The delay between the appearance of the go signal and the appearance of the stop signal is known at the stop-signal delay (SSD). This interval was varied in order to identify, for each participant, the offset at which a response was successfully inhibited more often than not. This was done by setting the SSD to a specific value (i.e., 250 ms), which was then constantly adjusted after stop-signal trials depending on whether the prepotent response was inhibited: When inhibition was successful, SSD increased by 50 ms; when inhibition was unsuccessful, SSD decreased by 50 ms. This made it possible to assign each participant a stop-signal reaction time (SSRT), reflecting how long it took a person to execute an inhibitory response to a prepotent action impulse. A longer SSRT indicated that it took longer to inhibit their response, indicating poorer behavioral inhibition (i.e., greater impulsivity).

## 3. Results

### 3.1. Pilot Study

The only significant correlations found between the time perspective measures and the eating-related variables were that the Present Hedonism subscale of the ZTPI was positively correlated with BMI (*r* = 0.24, *p* = 0.02), and the CFC—Immediate subscale was positively correlated with unhealthy eating (*r* = 0.22, *p* = 0.04), and negatively correlated with healthy eating (*r* = −0.26, *p* = 0.01). Based on these results, in a follow-up we focused on manipulating immediate versus future time perspective, and investigated CFC as a possible moderator.

### 3.2. Experiment 1

The descriptive statistics of the participants are presented in Table 1. There were no differences in hunger and mood (*F* (62) < 1, *p >* 0.88) and mood (*F* (62) < 2.25, *p* > 0.11) between the conditions.

To examine which factors best predicted the quantity of Maltesers and muesli consumed, a hierarchical linear regression was conducted for each dependent variable, with nested orthogonal condition contrasts (Time (present and future) conditions (1, 1) vs. Control (−2); and Present (1) vs. Future condition (−1)), and the moderators of BMI, restrained eating, and CFC mean score. The continuous variables were mean-centered. Analyses with positive and negative affect, and level of hunger as additional predictors and moderators revealed that these variables did not interact with any of the higher order significant effects, *t* (47) < 1.55, *p* > 0.12 for Maltesers, and *t* (47) > −1.43, *p* > 0.16 for muesli, and so the analysis was collapsed across all levels of these variables. Table 2 depicts the standardized regression coefficients of these analyses.

#### 3.2.1. Malteser Consumption

The more future-oriented participants were, the more Maltesers they ate, *t* (59) = 2.03, 95% CI (0.16, 2.21), *p* = 0.047. This effect interacted with the Future versus Present time perspective conditions, *t* (53) = 2.28, 95% CI (0.144, 2.23), *p* = 0.03.

Amongst the more present-oriented participants, those in the present condition tended to eat more than those in the future condition, and this pattern was reversed among more future-oriented participants. However, simple slopes analyses showed that these differences were not significant at 1 *SD* below and 1 *SD* above the mean, *b* = 1.68, *t* (61) = 1.102, *p* = 0.28 and *b* = −0.09, *t* (61) = −0.06, *p* = 0.95, respectively.

**Figure 1:**
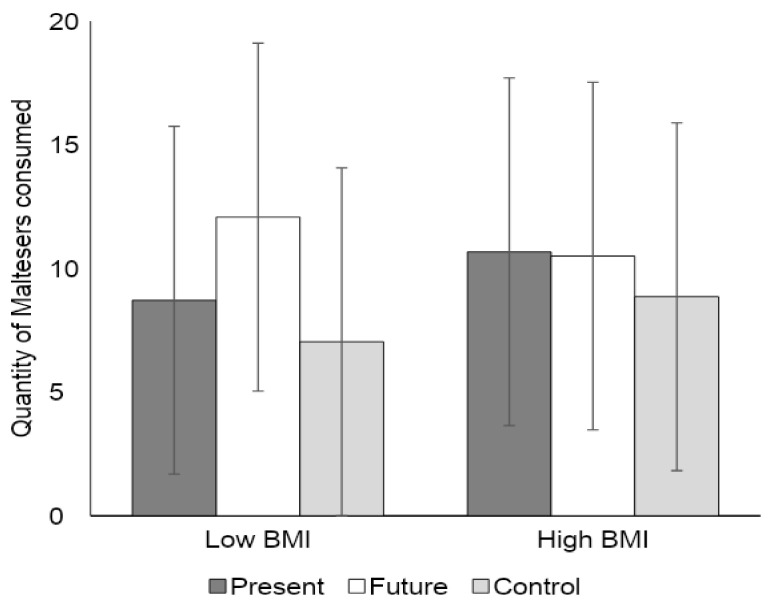
Quantity of Maltesers consumed at low (1 *SD* below the mean) and high (1 *SD* above the mean) levels of BMI for each condition in Experiment 1. The error bars represent the prediction intervals.

There was a significant interaction between BMI and the Future versus Present condition, *t* (53) = −2.28, 95% CI (−1.66, −0.12), *p* = 0.03). Figure 1 shows the quantity of Maltesers consumed at low (1 *SD* below the mean) and high (1 *SD* above the mean) levels of BMI for each condition. Amongst participants with relatively low BMI, those in the future condition consumed more Maltesers than those in the present condition. However, this effect was not significant, *b* = 1.68, *t* (61) = 1.18, *p* = 0.25. This pattern was reversed amongst participants with a higher BMI, but again, this effect was not significant, *b* = −0.09, *t* (61) = -0.06, *p* = 0.95.

There was also a significant interaction between restrained eating and the experimental versus control conditions, *t* (53) = 2.41, 95% CI (0.37, 3.96), *p* = 0.02. High restrained eaters consumed more Maltesers in the time conditions than the control condition, *b* = 3.01, *t* (61) = 2.67, *p* = 0.01. Although the pattern of means was reversed amongst low restrained eaters, this difference was not significant, *b* = −1.31, *t* (61) = −1.03, *p* = 0.31.

There was also a trend towards an interaction between restrained eating and the future versus present conditions, *t* (59) = 1.92, 95% (−0.13, 5.72), *p* = 0.06. Amongst high restrained eaters, there was a trend towards greater consumption of Maltesers in the future than the present condition, *b* = 3.59, *t* (61) = 1.70, *p* = 0.10. Although the pattern of means was reversed amongst low restrained eaters, this effect was not significant, *b* = −2.00, *t* (61) = −1.03, *p* = 0.30.

#### 3.2.2. Muesli Consumption

As in with the consumption of Maltesers, the more future-oriented participants were, the more muesli they ate, *t* (59) = 2.80, 95% CI (0.36, 2.15), *p* = 0.01. The only other significant effect was an interaction between restrained eating and the contrast between the experimental time conditions combined versus the control condition, *t* (53) = 3.33, 95% CI (1.02, 4.12), *p* = 0.002. Simple slopes analyses revealed that, at low levels of dietary restraint, participants in the control condition ate more muesli than those in the experimental time conditions, whereas this pattern was reversed at high levels of dietary restraint.

### 3.3. Experiment 2

Table 3 shows the descriptive statistics of Experiment 2. There were no differences in hunger and mood (*F* ≤ 1.65, *p* ≥ 0.2) between the conditions. As α = 0.47 with the two hunger items, we only included the reported level of hunger, and not the reported level of fullness, in the analyses. The α for the restrained eating scale was 0.53 with all five questions, so Q4 (“Do you watch exactly what you eat?”) was removed in order to improve internal consistency (see below).

We examined correlations between the variables of interest (see Table 4). Amount of Maltesers consumed did not correlate with any of the variables except the amount of muesli that was eaten (*r* = 0.369, *p* < 0.001). Answers on the stop-signal task and money discounting did not correlate with any of the variables. However, lower discounting of food was associated with higher dietary restraint. Moreover, higher levels of dietary restraint were associated with higher BMI and consideration of future consequences.

As in Experiment 1, a hierarchical linear regression was conducted for each dependent variable, with nested orthogonal condition contrasts and the moderators of BMI, restrained eating (α = 0.79), and CFC mean score (α = 0.80; CFC-Future subscale α = 0.76, CFC-Immediate subscale α = 0.86). The continuous variables were mean centered. Analyses with affect (α = 0.83 for positive affect, α = 0.77 for negative affect), and level of hunger as additional predictors and moderators revealed that these variables did not interact with any of the higher-order significant effects (*t* (135) > −1.86, *p* > 0.07 for Maltesers, and *t* (135) > −1.94, *p* > 0.06 for muesli). Table 5 depicts the standardized regression coefficients of these analyses. 

#### 3.3.1. Malteser Consumption

**Figure 2:**
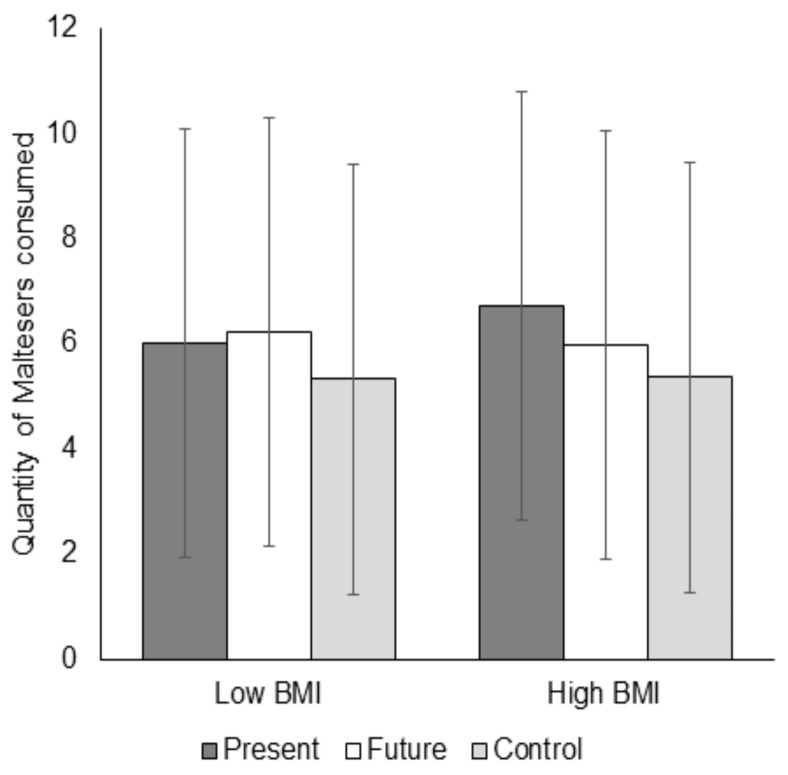
Quantity of Maltesers consumed at low (1 *SD* below the mean) and high (1 *SD* above the mean) levels of BMI for each condition in Experiment 2. The error bars represent the prediction intervals.

The only significant effect found was an interaction between BMI and the Future versus Present condition, *t* (130) = −1.99, 95% CI (−0.42, −0.00), *p* = 0.049. Figure 2 shows the quantity of Maltesers consumed at low (1 *SD* below the mean) and high (1 *SD* above the mean) levels of BMI for each condition. Simple slopes analyses revealed that, although participants with a low BMI tended to eat more in the future than the present condition, and that this pattern of effects was reversed for those with a higher BMI, these differences were not statistically significant at 1 *SD* above and below the mean, *b* = 0.07, *t* (132) = 0.16, *p* = 0.88 and *b* = −0.35, *t* (132) = −0.77, *p* = 0.44, respectively.

#### 3.3.2. Muesli Consumption

No significant effects were found for muesli consumption *t* (130) < −1.22, *p* > 0.18.

#### 3.3.3. Mediation by Impulsivity

We then tested whether impulsivity mediated the influence of time perspective (Future vs. Present) on Malteser intake for both measures of impulsivity.

#### 3.3.4. Delay Discounting

We first tested whether time perspective had an influence on money or food discounting with stepwise regressions. First, we tested a model that included the time perspective contrasts alongside the variables restrained eating, BMI, and CFC, and in a second step, we added the interaction between the condition contrasts and each of the other variables.

For money, step 1 revealed that higher BMI predicted more money discounting, *b* = 0.04, *t* (393) = 2.38, *p* = 0.018, 95% CI (0.01, 0.08), and that being more future-oriented was associated with lower money discounting, *b* = −0.11, *t* (393) = −2.76, *p* = 0.006, 95% CI (−0.19, −0.03). In step 2, there was an effect of Time perspective (Future vs. Present) × Restraint, *b* = 0.21, *t* (397) = 2.16, *p* = 0.032, 95% CI (0.02, 0.41), indicating that restraint is associated with more discounting in particular in the Present (vs. Future) condition. Simple slopes analyses revealed that, for those with low restraint (1 *SD* below the mean), there was no influence of time perspective (Future vs. Present) on money discounting, *b* = −0.18, *p* = 0.510. For participants with high restraint (1 *SD* above the mean), there was an influence of time perspective (Future vs. Present): *b* = 0.32, *t* (387) = 2.30, *p* = 0.022, 95% CI (0.05, 0.59), indicating that high-restraint participants discount money less when writing about their future (compared to their present).

For food, in step 1, lower levels of restrained eating were associated with more food discounting, *b* = −0.16, *t* (393) = −2.36, *p* = 0.019, 95% CI (−0.29, −0.03), although the overall model did not reach significance, *F* (5, 393) = 1.69, *p* = 0.14. Including the interaction coefficients in step 2 revealed two significant interactions between Time perspective × BMI. More specifically, BMI interacted with the contrast Time vs. Control, *b* = 0.03, *t* (387) = 2.66, *p* = 0.008, 95% CI (0.01, 0.05). Again, the overall model was not significant, *F* (11, 387) = 1.56, *p* = 0.107.

Simple slopes analyses indicated that, for those with low BMI (1 *SD* below the mean), there was no influence of time perspective on food discounting, *p* = 0.171. However, for participants with high BMI (1 *SD* above the mean of BMI), there was a significant influence of time perspective (Time vs. Control) on food discounting, *b* = 0.17, *t* (387) = 2.47, *p* = 0.014, 95% CI (0.03, 0.31). Participants with high BMI discounted more food when they wrote about what their lives were like in the present, or would be like in the future, compared to when they wrote about the latest film they watched.

We then tested the influence of delay discounting on food consumption. For money and food separately, we tested whether discounting was associated with Malteser consumption, controlling for the influence of Time perspective (Future vs. Present) × BMI, control variables, as well as the interaction between BMI and the discounting variable. For both money and food discounting, the influence of Time perspective (Future vs. Present) × BMI remained significant (*b* = 0.18, *p* = 0.004 and *b* = 0.24, *p* < 0.001, respectively), indicating that delay discounting of money or food does not mediate the influence of present versus future time perspective on Malteser intake.

#### 3.3.5. Stop-Signal Task

Averaged over all subjects, mean reaction time on the go task when no stop signals were presented (i.e., the mean go signal reaction time) was 768.26 ms (*SD* = 10767 ms) and mean accuracy was 68.05% (*SD* = 0.15). The mean SSRT was 101.70 ms (*SD* = 1568.46 ms). Higher scores reflect greater impulsivity. We first examined whether time perspective had an influence on reaction times and accuracy on the stop-signal task. Following the same analyses that were conducted for discounting above, we conducted two separate regression with reaction times as the outcome in one regression, and accuracy as the outcome in the other regression. 

Concerning accuracy, there were no significant influences of Time perspective, the other variables, nor their interactions on delay discounting. For reaction time, however, there was a significant influence of CFC × Time perspective (Time vs. Control), *b* = −0.34, *t* (130) = −3.53, *p* = 0.001, 95% CI (326.40, −91.72). Participants who considered future consequences were *less* impulsive (had lower scores) in the time conditions than the control condition, whereas those who were more oriented towards the present were more impulsive (had higher scores) in the time conditions than the control condition. Simple slopes analyses revealed that, when participants had a CFC score 1 *SD* below the mean (i.e., they were more focused on the present), they tended to have lower SSRT scores in the time conditions than the control condition, suggesting that they were more impulsive in the control condition, *b* = 626.70, *t* (132) = 4.32, *p* < 0.01). This pattern of effects was reversed for those who had a higher CFC score (i.e., they were more focused on the future), although the difference between the time conditions was not quite statistically significant at 1 *SD* above the mean, *b* = −209.54, *t* (132) = −1.81, *p* = 0.07.

We then tested whether RT was associated with Malteser consumption, controlling for the influence of Time perspective (Future vs. Present) × BMI, control variables, as well as the interaction between BMI and reaction time. The influence of Time perspective (Future vs. Present) × BMI remained marginally significant, *b* = −0.19, *t* (132) = 0.16, *p* = 0.058, 95% CI (−0.436, −0.008), suggesting that impulsivity does not mediate its influence on Malteser intake.

## 4. Discussion

### 4.1. Experiment 1

In Experiment 1, the amount of unhealthy food participants consumed interacted with participant BMI, dietary restraint, and their individual time preference. If we consider that unhealthier eating behavior tends to be correlated with a higher BMI [4], and a more present time-orientation [7], it would appear that, compared to the present condition, those in the future condition consume less unhealthy food than they would normally, which is somewhat consistent with our hypotheses. However, this interpretation should be made with caution, given that we did not find any main effect of BMI or dietary restraint in this study, and that we found that participants who had a greater future time preference actually ate more unhealthy and healthy food.

There was also an interaction between restrained eating and the contrast between the two experimental time conditions versus the control condition. For both chocolate and muesli, high-restrained eaters tended to consume more in the time conditions than in the control condition, whereas this pattern tended to reverse amongst low-restrained eaters. This suggests that high-restrained eaters may eat more when their attention is drawn towards themselves, as was the case in the time conditions.

The results of Experiment 1 suggest that thinking about the future can reduce the consumption of unhealthy food in those who may be more prone to eating unhealthily (i.e., those with a higher BMI, those who do not try to restrict their caloric intake, and those who are more oriented towards the present). A possible reason why this may occur is that thinking about the future reduces impulsivity: Dassen and colleagues [23] have shown that thinking about an upcoming food-related episode reduces monetary and food discounting, which are measures of impulsivity. The authors did not find a relationship between either type of discounting and food consumption. However, given that our manipulation is slightly different from theirs, we thought it may be more sensitive to mediation effects, and tested this in Experiment 2.

### 4.2. Experiment 2

Experiment 2 replicated the finding of Experiment 1 that thinking about the future is associated with lower consumption of unhealthy food in individuals with high BMI. However, we did not replicate this finding for participants with low restraint or with a present time orientation.

Contrary to our hypotheses, the two measures of impulsivity assessed, delay discounting and inhibition, did not mediate the relationship between time perspective and food intake. Moreover, replicating the finding of Dassen and colleagues [23], unhealthy food intake was not associated with delay discounting, nor our additional measure of impulsivity, i.e., inhibition as measured by the Stop-Signal Task.

### 4.3. General Discussion

In two experiments, it was tested whether thinking about the future versus the present, or a situation unrelated to time, impacts the amount of unhealthy food individuals consume. In both experiments, we found that time perspective interacted with individual factors. In Experiment 1, individuals consumed less chocolate when they thought about the future, if they had higher BMI, lower dietary restraint, or were in general more present-oriented. In Experiment 2, we replicated this effect for high-BMI participants, but not for the other individual factors. In this experiment we also tested whether impulsivity mediated the influence of time perspective on chocolate intake, and found that this was not the case.

The two experiments in this study suggest that thinking about the future may be an effective strategy to reduce unhealthy food intake in individuals more prone to this behavior (e.g., with a higher BMI, lower dietary restraint, and who are less considerate of future consequences). This result is consistent with previous findings showing that, for instance, individuals with a higher BMI tend to be less considerate of future consequences [21]. Consequently, prompting them to focus on their future may activate future goals and make them more considerate of these goals in their current decision-making processes. Such a prompt may be less effective for individuals with a lower BMI, higher restraint, and who are more future-oriented, because they already tend to consider the future consequences of their present actions.

Alternatively, unhealthy food intake may only be affected by time orientation in individuals who are motivated to consume the food [40]. Contrary to the experiment by Dassen and colleagues [23], participants in our study were not asked to fast prior to the experiment, which could have led to them being overall less motivated to consume the unhealthy food. This offers a possible explanation for why thinking about the present resulted in higher unhealthy food intake only for those most motivated to obtain the food: those with a higher BMI, lower dietary restraint, and who are more present-oriented.

Contrary to our expectations, impulsivity did not play a role in the relationship between time perspective and unhealthy food intake. However, participants with a higher BMI or who were more present-oriented tended to be more impulsive when writing about their present or future than when writing about the last movie they watched. Possibly, focusing on oneself (vs. a topic unrelated to the self) leads to engagement in unhealthy behaviors in individuals who tend to be more prone to unhealthy eating. This is consistent with the theory that binge eaters engage in disinhibited eating in order to escape from distressing self-awareness [41].

Moreover, our impulsivity measures did not correlate with the amount of food participants consumed. This finding is consistent with previous work such as that of Dassen [23], as well as a study in which there was no effect of time perspective on delay discounting or food consumption in a within-participant design, where participants engaged in episodic future thinking in 1 week and episodic recent thinking in another week [42]. While other studies have found associations between time perspective and BMI or impulsivity, the relationship between these three variables remains unclear. A literature review by Teuscher and Mitchell [13] revealed a stronger link between obesity and delay discounting than between obesity and time perspective. Possibly, unhealthy food intake may also have diverging associations with time perspective and delay discounting.

Additionally, a third variable may be at play, such as food reinforcement, which describes how hard someone is willing to work to obtain a desired food [43]. Food reinforcement has been found to interact with delay discounting in predicting food intake [44]. This resonates with the abovementioned idea that time perspective may only affect the food intake of individuals who are motivated to obtain the food, i.e., they have a higher reward sensitivity. A possible way to test this would be to manipulate hunger, or motivation for food, and see how time perspective affects impulsivity and food intake between differing conditions of food motivation.

### 4.4. Limitations and Future Perspectives

Our study sample consisted of young adults: an age group that has been associated with lower consideration of future consequences [21], and more impulsive tendencies [45]. This could have made them less sensitive to our time perspective manipulation than the general population. Nevertheless, a recent study showed that episodic imagining had, in fact, a stronger effect on delay discounting in younger adults than older adults [46]. Moreover, previous studies found effects of time perspective on eating in young samples (see [47] for a review).

In our second experiment, we measured our mediator, the impulsivity measures, after the dependent variable, consumption during the taste test. This means that consumption may have influenced the behavior that was captured by the impulsivity measures, providing a tentative explanation for why we find no mediation. Others have suggested a similar negative spill-over effect of self-control exertion from one domain or time point to another [48,49]. In a future study, counterbalancing the impulsivity and consumption measures could clarify the association between time perspective, impulsivity, and unhealthy food intake.

In addition, the lack of correlation between our impulsivity measures reflects conceptual differences found in impulsivity research (e.g., see [50]). For instance, a recent meta-analysis demonstrated a significant relationship between BMI and money discounting but not between BMI and the Stop-Signal Task [51]. Different components of impulsivity may be at play in translating time-related effects on unhealthy eating behaviors.

The presents results are in line with those of those of another study by Segovia, Palma, and Nayga [52] that found that thinking about the future reduces unhealthy snack choice amongst those who are obese only, but not those of a lower BMI. However, when instructed to think about the future, participants in that study were presented with modified images of themselves as being weight-increased and weight-reduced. Thus, it may be that people who were obese felt differently about their weight-increased and weight-reduced images than those of a lower BMI. As such, future research could assess whether the effect of thinking about the future on unhealthy eating might be mediated by the content/quality of people’s thoughts.

Notwithstanding the need to further explore the relationship between time perspective, impulsivity, and unhealthy food intake, our results indicate that changing time perspective may be a promising intervention to reduce unhealthy food consumption in vulnerable subgroups (e.g., individuals with high BMI) and in individuals who are motivated to reduce their caloric intake (see also [53]).

## 5. Conclusions

Across two experiments, the effect of thinking about the future versus the present interacted with participants’ body mass index (BMI) to influence their consumption of unhealthy food, but no reliable effects were found for the consumption of healthy food. Among individuals with a higher BMI, thinking about their lives in the future resulted in lower consumption of the unhealthy food compared to thinking about their lives in the present. However, this effect was reversed for those with a lower BMI. This suggests that thinking about the future can reduce unhealthy eating among heavier people.

## Figures and Tables

**Table 1:** Descriptive statistics of Experiment 1.

	Overall Mean (*n* = 65)	Present (*n* = 23)	Future (*n* = 21)	Control (*n* = 21)
Positive Affect	3.16 (0.65)	3.16 (0.69)	3.22 (0.64)	3.10 (0.62)
Negative Affect	1.47 (0.48)	1.65 (0.59)	1.22 (0.21)	1.52 (0.47)
Hunger	3.74 (1.15)	3.83 (1.21)	3.71 (1.02)	3.67 (1.26)
CFC-Immediate	3.45 (1.25)	3.94 (1.36)	3.12 (1.22)	3.24 (1.01)
CFC-Future	4.82 (1.23)	4.53 (1.29)	5.18 (1.02)	4.77 (1.30)
CFC-Mean	1.37 (2.14)	0.59 (2.10)	2.07 (2.10)	1.52 (2.04)
Restrained Eating	2.42 (0.88)	2.35 (0.76)	2.28 (0.94)	2.66 (0.93)
BMI	21.95 (3.03)	22.03 (3.27)	21.51 (3.03)	22.29 (2.86)
Quantity of Maltesers eaten (in grams)	10.05 (9.03)	9.30 (8.39)	12.38(10.67)	8.52 (7.85)
Quantity of muesli eaten (in grams)	5.52 (7.64)	3.59 (2.75)	7.05 (6.58)	6.12 (11.31)

Note. *SD*s are in parentheses. BMI: body mass index. CFC: consideration of future consequences.

**Table 2:** Standardized regression coefficients in Experiment 1 for amount of Maltesers and muesli consumed (unstandardized coefficients, standard error in parentheses).

		Maltesers *B*	Muesli *B*
Step 1	BMI	0.12 (0.37, 0.38)	0.01 (0.28, 0.31)
	Restraint	−0.05 (−0.54, 1.34)	−0.1–0.17 (−1.46, 1.10)
	CFC	0.26 (1.11, 0.55) *	0.35 (1.25, 0.45) **
	Time vs. Control	0.13 (0.85, 0.80)	−0.06 (−0.30, 0.65)
	Future vs. Present	0.07 (0.80, 1.40)	0.09 (0.83, 1.15)
	*F*	1.45	2.38 *
	*R* ^2^	0.11	0.06
Step 2	BMI	0.15 (0.44, 0.35)	0.17 (0.44, 0.31)
	Restraint	−0.10 (−1.03, 1.23)	−0.20 (−1.77, 1.06)
	CFC	0.26 (1.10, 0.49) *	0.31 (1.11, 0.42)
	Time vs. Control	0.03 (0.17, 0.72)	−0.14 (−0.73, 0.62)
	Future vs. Present	0.10 (1.06, 1.26)	0.15 (1.41, 1.09)
	BMI × Time vs. Control	−0.12 (−0.27, 0.28)	−0.22 (−0.42, 0.24)
	BMI × Future vs. Present	−0.25 (−0.88, 0.39) *	−0.04 (−0.12, 0.33)
	Restraint × Time vs. Control	0.30 (2.16, 0.89) *	0.42 (2.57, 0.77) **
	Restrained × Future vs. Present	0.22 (2.79, 1.46) ^+^	0.07 (0.75, 1.26)
	CFC × Time vs. Control	0.01 (0.04, 0.36)	−0.14 (−0.36, 0.31)
	CFC × Future vs. Present	0.27 (1.39, 0.59) *	0.15 (0.65, 0.50)
	*F*	3.15 **	2.84 **
	*R* ^2^	0.40	0.37

Note. ^+^ *p =* 0.06, * *p* < 0.05, ** *p* < 0.01. Including the interaction term in Step 2 means that the associated main effects show the effect of the predictor when the value of the other predictors are set at 0 or at their mean (for centered predictors).

**Table 3:** Descriptive statistics of Experiment 2 (SD in parentheses).

	Overall Mean (*N* = 136)	Present (*n* = 51)	Future (*n* = 45)	Control (*n* = 40)
Positive Affect	3.01 (0.64)	3.08 (0.72)	2.99 (0.56)	2.93 (0.61)
Negative Affect	1.40 (0.41)	1.47 (0.46)	1.39 (0.41)	1.34 (0.34)
Hunger	1.91 (1.16)	2.04 (1.15)	1.73 (1.21)	1.95 (1.13)
CFC-Immediate	3.50 (1.23)	3.57 (1.07)	3.43 (1.43)	3.50 (1.19)
CFC-Future	4.77 (1.06)	4.96 (0.99)	4.56 (1.23)	4.75 (0.93)
CFC-Mean	1.26 (1.93)	1.39 (1.76)	1.18 (2.21)	1.26 (1.83)
Restrained Eating	2.68 (0.94)	2.80 (0.95)	2.48 (0.99)	2.77 (0.86)
BMI	22.65 (4.36)	23.51 (4.75)	22.27 (3.96)	22.04 (4.08)
Quantity of Maltesers eaten (in grams)	5.98 (4.28)	6.43 (4.75)	6.07 (4.72)	5.30 (3.78)
Quantity of muesli eaten (in grams)	3.15 (2.52)	3.39 (2.59)	3.27 (2.59)	2.70 (2.36)
Budget	289.63 (384.76)	341.57 (536.62)	226.05 (232.83)	291.75 (263.25)
Stop-Signal RT (ms)	101.70 (1586.46)	218.75(150.89)	257.70 (148.05)	−223.77 (290.30)
Stop-Signal ACC	0.68 (0.15)	0.66(0.17)	0.67 (0.12)	0.71 (0.15)
Money discounting	0.038 (0.050)	0.039 (0.056)	0.032 (0.037)	0.044 (0.055)
Food discounting	0.231 (0.190)	0.220 (0.187)	0.254 (0.204)	0.218 (0.182)

**Table 4:** Correlations coefficients between variables in Experiment 2.

	1	2	3	4	5	6	7	8
1. Malteser consumption								
2. Muesli consumption	0.370 **							
3. Money discounting	0.079	0.128						
4. Food discounting	0.066	0.080	0.135					
5. Stop-Signal RT	0.011	−0.011	−0.037	−0.103				
6. Stop-Signal ACC	−0.121	−0.187 *	−0.134	0.103	−0.037			
7. BMI	0.094	0.118	0.126	−0.046	0.051	−0.076		
8. CFC	0.024	0.010	−0.127	−0.054	0.156	−0.086	0.121	
9. Restraint	0.005	0.055	0.054	−0.178 *	−0.040	0.021	0.250 *	0.216 *

Note. * *p* < 0.05, ** *p* < 0.001.

**Table 5:** Standardized regression coefficients in Experiment 2 for amount of Maltesers and muesli consumed (unstandardized coefficients, standard error in parentheses).

		Maltesers *B*	Muesli *B*	Stop-Signal Task RT *B*	Stop-Signal Task ACC *B*	Money Discounting	Food Discounting
Step 1	BMI	0.08 (0.08, 0.09)	0.10 (0.06, 0.05)	0.04 (13.55, 33.40)	−0.06 (−0.00, 0.00)	0.12 (0.04, 0.02) *	<0.001 (<0.001, 0.01)
	Restraint	−0.02 (−0.08, 0.42)	0.04 (0.11, 0.25)	−0.07 (−116.72, 157.65)	0.04 (0.01, 0.02)	0.04 (0.06, 0.09)	−0.13 (−0.16, 0.07) *
	CFC	0.02 (0.04, 0.20)	−0.01 (−0.02, 0.12)	0.16 (130.45, 74.31)	−0.08 (−0.01, 0.01)	−0.14 (−0.11, 0.04) **	−0.008 (−0.005, 0.03)
	Time vs. Control	0.09 (0.29, 0.27)	0.108 (0.198, 0.16)	0.12 (138.79, 104.79)	−0.13 (−0.014, 0.01)	−0.08 (−0.09, 0.06)	0.03 (0.03, 0.04)
	Future vs. Present	−0.027 (0.289, 0.27)	−0.004 (−0.012, 0.26)	0.02 (32.72, 170.07)	0.00 (0.01, 0.02)	0.05 (0.09, 0.09)	−0.04 (−0.06, 0.07)
	*F*	0.50	0.70	1.14	0.75	3.30 **	1.69
	*R* ^2^	0.02	0.03	0.05	0.03	0.04	0.02
Step 2	BMI	0.03 (0.03, 0.093)	0.12 (0.07, 0.06)	0.05 (17.76, 33.67)	−0.03 (−0.00, 0.02)	0.10 (0.04, 0.02)	−0.01 (−0.003, 0.01)
	Restraint	−0.00(−0.01, 0.44)	0.05 (0.12, 0.26)	−0.15 (−251.03, 160.47)	0.07 (0.012, 0.02)	0.03 (0.05, 0.09)	−0.11 (−0.14, 0.07)
	CFC	0.01 (0.03, 0.21)	0.01 (0.01,0.12)	0.26 (208.58, 76.81)	−0.09 (−0.01, 0.01)	−0.13 (−0.10, 0.04)	<0.001 (<0.001, 0.03)
	Time vs. Control	0.08 (0.26, 0.28)	0.11 (0.20, 0.16)	0.09 (101.67, 102.78)	−0.11 (−0.01, 0.01)	−0.10 (−0.12, 0.06)	0.05 (0.04, 0.04)
	Future vs. Present	−0.03 (−0.16, 0.45)	−0.01 (−0.02 ,0.27)	0.01 (14.66, 165.41)	0.02 (.04, 0.02)	0.06 (0.12, 0.09)	−0.05 (−0.07, 0.07)
	BMI × Time vs. Control	0.07 (0.05, 0.07)	−0.04 (−0.018, 0.04)	−0.08 (−21.84, 25.08)	−0.01 (0.00, 0.00)	−0.05 (−0.01, 0.01)	0.14 (0.03, 0.01) **
	BMI × Future vs. Present	−0.18 (−0.21, 0.11) *	0.13 (0.09, 0.06)	0.01 (2.53, 38.90)	0.17 (0.01, 0.00)	0.07 (0.03, 0.02)	−0.08 (−0.03, 0.02)
	Restraint × Time vs. Control	−0.06 (−0.21, 0.34)	0.02 (0.04,0.20)	0.18 (225.55, 125.04)	−0.07 (−0.01, 0.01)	0.002 (0.002, 0.07)	−0.05 (−0.04, 0.05)
	Restrained × Future vs. Present	−0.12 (−0.65, 0.47)	0.020 (0.06, 0.28)	0.011 (20.51, 174.21)	−0.08 (−0.01, 0.02)	0.11 (0.21, 0.09) *	0.04 (0.06, 0.08)
	CFC × Time vs. Control	−0.05 (−0.09, 0.16)	0.050 (0.05, 0.09)	−0.34 (−209.06, −59.22) **	0.16 (0.01, 0.01)	−0.02 (−0.01, 0.03)	0.008 (0.004, 0.03)
	CFC × Future vs. Present	0.01 (0.02, 0.23)	−0.121 (−0.18, 0.14)	−0.01 (−6.96, 84.73)	−0.04 (−0.00, 0.01)	0.04 (0.04, 0.05)	−0.002 (−0.001, 0.04)
	*F*	1.05	0.68	1.86 *	0.86	2.45 **	1.56
	*R* ^2^	0.09	0.06	0.16	0.08	0.07	0.04

Note. * *p* ≤ 0.05, ** *p* < 0.01. Including the interaction term in Step 2 means that the associated main effects show the effect of the predictor when the value of the other predictors are set at 0 or at their mean (for centered predictors). For money and food discounting, reward Size was added in the model as a control variable.
